# Why health information technology safety problems remain invisible

**DOI:** 10.3389/fdgth.2026.1785141

**Published:** 2026-05-15

**Authors:** Md Shafiqur Rahman Jabin

**Affiliations:** 1 Department of Medicine and Optometry, Linnaeus University, Kalmar, Sweden; 2 Faculty of Health and Social Care, University of Bradford, Bradford, United Kingdom

**Keywords:** automation bias, health information technology safety, health IT, incident reporting systems, patient safety, safety culture, sociotechnical systems

## Introduction: The paradox of “safe” digital healthcare

Digital health technologies are widely promoted as instruments of safety. Electronic health records, prescribing systems, and tools promise improved legibility, standardisation, traceability, and control (1–3). Compared with paper-based care, digital systems are often assumed to reduce error by constraining unsafe actions and embedding safeguards into routine work (4). As a result, healthcare organisations frequently interpret the absence of major system failures as evidence that digital safety risks are under control (5, 6).

Yet this confidence sits uneasily alongside recurring reports of serious, sometimes catastrophic, health information technology (HIT)–related incidents. When such events surface, they are often described as unexpected, difficult to explain, or “out of the blue.” This raises a fundamental question: if digital systems are safer, why do their failures continue to surprise us? (5–7).

This article advances a simple but uncomfortable claim: *many of the most significant safety risks associated with digital health technologies are not rare, but invisible.* Rather than manifesting as obvious breakdowns, these risks often remain hidden within everyday work, masked by automation, adaptation, and organisational assumptions about how digital systems function (8, 9). Understanding digital safety, therefore, requires not only preventing failure but learning to recognise what currently goes unseen (8–10).

In this context, “invisibility” refers not simply to the absence of observable failure, but to the systematic difficulty of recognising how risks emerge, propagate, and are normalised within digitally mediated care. This perspective aligns with sociotechnical and Safety-II approaches, which emphasise that system performance must be understood in terms of everyday functioning rather than isolated failures (9, 11, 12). By focusing on what is not readily seen, this paper shifts attention from discrete adverse events to the underlying conditions that enable them.

## Invisibility as a defining feature of digital risk

Invisibility in digital healthcare does not mean that nothing is happening. Instead, it refers to the way risks can exist without producing immediate, recognisable signals of harm. Digital hazards may accumulate slowly, propagate across systems, or affect decision-making indirectly. They may be detected only after downstream consequences emerge, sometimes long after the original conditions were established (12–14).

This contrasts with many traditional clinical incidents, which tend to be temporally bounded and directly observable (14). A medication error, a procedural complication, or a diagnostic delay often presents as a discrete event with a relatively clear temporal relationship between action and outcome (15). Digital safety problems, by contrast, frequently unfold as processes rather than events. Data may be wrong, incomplete, delayed, or misleading, yet care continues, sometimes successfully, until a tipping point is reached (15–17).

**Figure 1:**
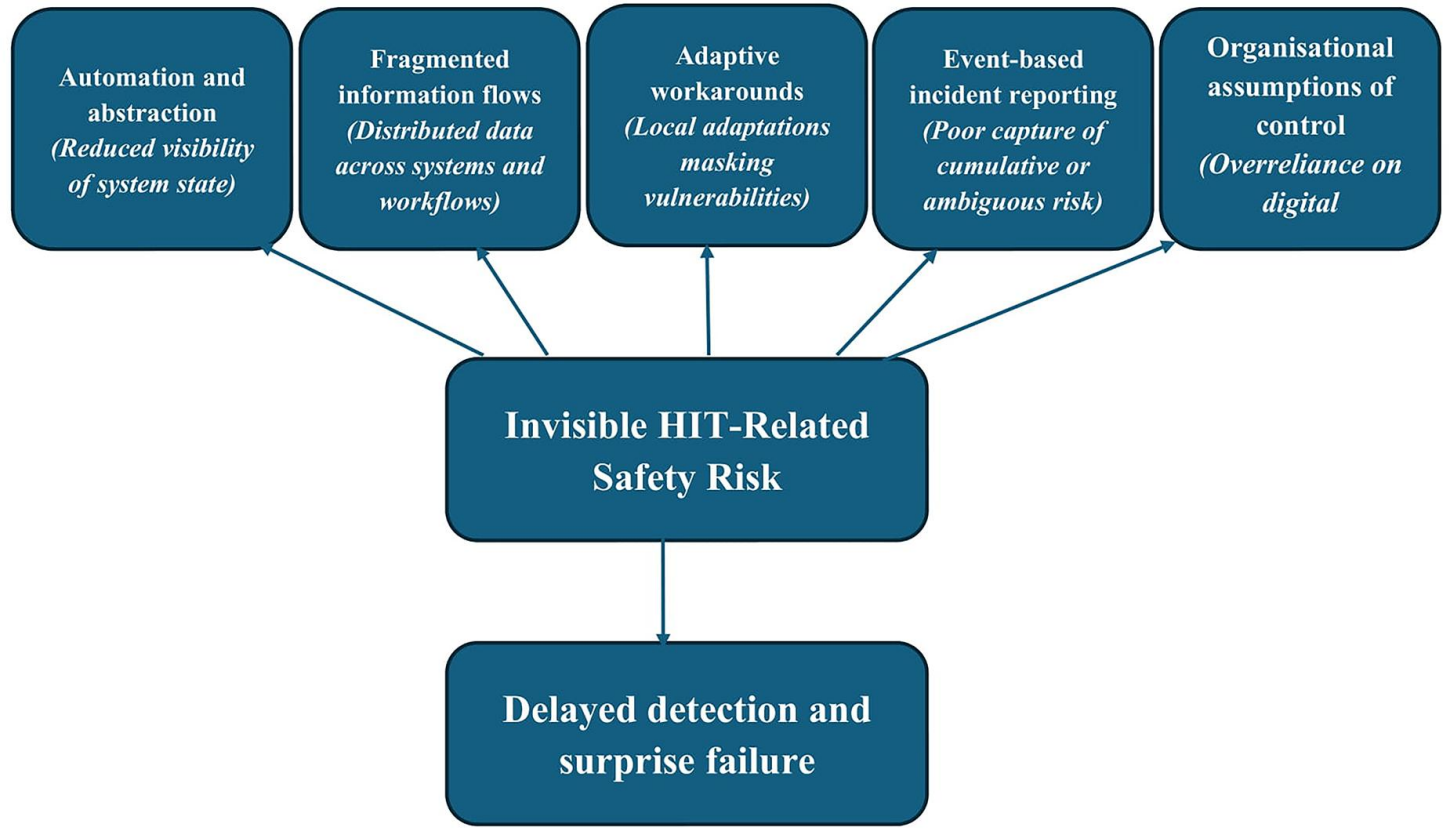
Mechanism contributing to the invisibility of HIT-related safety risk. In digitally mediated healthcare, safety risks often remain hidden due to the combine effects of automation and abstraction, fragmented information flows, adaptive workarounds, event-based incident reporting, and organizational assumptions of control. These mechanisms act to reduce the visibility of system behaviour contributing to delayed recognition of risk and, ultimately, to surprise failures when harm becomes apparent.

Crucially, invisibility is not accidental. It is a structural feature of how digital systems are designed, implemented, and used. Digital technologies reorganise work in ways that reduce the visibility of underlying processes while increasing reliance on system outputs. In doing so, they change not only how care is delivered, but how risk is perceived (18, 19). The mechanisms through which digital safety risks remain invisible are summarised in Figure 1.

For example, a delay in the propagation of laboratory results across interconnected systems may not be immediately visible to clinicians, particularly when interfaces present partial or cached data. Care may proceed without interruption, and only later, when clinical deterioration occurs, does the underlying data inconsistency become apparent (20, 21). Such scenarios illustrate how digital risks unfold over time, often without clear signals at the point of care.

### Mechanisms that render digital safety risk invisible

The invisibility of digital safety risk arises from a set of interrelated mechanisms embedded in the design, use, and governance of health information technologies. As illustrated in Figure 1, five key mechanisms contribute to this phenomenon: automation and abstraction, fragmented information flows, adaptive workarounds, event-based incident reporting, and organisational assumptions of control. Each mechanism reduces the visibility of system behaviour in different but reinforcing ways.

### Automation and abstraction

Automation plays a central role in making digital risk difficult to see. Automated functions remove tasks from direct human control, embedding assumptions about timing, accuracy, and relevance into system logic (22, 23). While this can reduce workload and variability, it also obscures the conditions under which outputs are generated. Clinicians interact with results, alerts, and summaries, but have limited insight into how these artefacts are produced or what has been filtered out along the way (24, 25).

Interface design further amplifies this abstraction. Digital systems present simplified representations of complex clinical and technical states, prioritising usability and efficiency over transparency. Information is segmented across screens, systems, and workflows, making it difficult to develop a holistic understanding of what the system “knows” at any given moment. As a result, clinicians may trust system outputs even when those outputs are incomplete, outdated, or contextually inappropriate (26, 27).

When failures occur under these conditions, they often appear as surprises rather than as predictable outcomes of degraded system performance. The absence of visible warning signs reinforces the belief that systems are functioning correctly, delaying recognition of emerging hazards (6, 28).

This dynamic is closely related to automation bias, where users tend to over-rely on system-generated outputs while under-scrutinising underlying processes (8). As visibility into system functioning decreases, trust in outputs may increase, even when those outputs are based on incomplete or contextually inappropriate data.

### Fragmented information flows

Fragmented information flows further contribute to the invisibility of digital risk. Clinical data are often distributed across multiple systems, interfaces, and workflows, each presenting only a partial view of the patient or process. As a result, no single actor has full visibility of the system state at any given time (29, 30). Important discrepancies, such as missing, delayed, or conflicting information, may therefore go unnoticed, particularly when systems do not support integrated or longitudinal views of care (21, 29, 30).

This fragmentation introduces both cognitive and organisational challenges. Clinicians may be required to navigate multiple interfaces, reconcile inconsistencies, and infer missing information under time pressure, increasing the likelihood that discrepancies remain unnoticed (27). In some cases, information may be present within the broader system but effectively invisible in practice because it is not accessible at the point of decision-making or is distributed across disconnected workflows (27, 31).

Under these conditions, important signals of emerging risk, such as delayed results, incomplete data, or conflicting information, may not be recognised. Instead, they are absorbed into routine work, allowing care to proceed while underlying system vulnerabilities remain hidden; harm is frequently attributed to individuals.

### Adaptive workarounds

Another powerful source of invisibility lies in everyday clinical adaptation. Healthcare work is characterised by continuous adjustment, as clinicians develop workarounds to cope with mismatches between system design and the realities of practice. In digital environments, these adaptations are often essential for maintaining efficiency and continuity of care (31, 32).

Workarounds are frequently interpreted as evidence of resilience. They demonstrate ingenuity, flexibility, and professional commitment. However, they also have a paradoxical effect: *successful adaptation can conceal underlying weaknesses in the system* (6, 26)*.* When clinicians compensate for design flaws, configuration problems, or poor integration, the system appears to function adequately, even though it relies on fragile and informal practices (33, 34).

Over time, such adaptations become normalised. Deviations from intended use are no longer recognised as signals of risk but as routine aspects of work. In this way, success masks failure, and the absence of incidents is taken as confirmation that no problem exists (35, 36). When harm eventually occurs, attention often shifts to the final action rather than the long-standing conditions that made the system vulnerable.

This normalisation of workarounds reflects a broader pattern described in safety science, in which deviations from intended processes become routine and are no longer recognised as sources of risk (37–40). In this way, adaptive behaviour contributes not only to system resilience but also to the concealment of underlying vulnerabilities.

### Event-based incident reporting

Incident reporting systems are a cornerstone of patient safety learning, yet they are poorly suited to capturing invisible digital risks. Most reporting systems were designed around assumptions of discrete events, identifiable errors, and observable harm (41). Digital safety problems often do not fit these templates (42).

Many HIT-related issues do not trigger reports because they are ambiguous, recurrent, or lack immediate consequences. Clinicians may experience frustration, uncertainty, or increased workload without perceiving these as reportable safety events. Others may not recognise digital anomalies as hazards at all, particularly when no patient harm is apparent (14).

Even when reports are submitted, existing classification schemes may fragment sociotechnical interactions into isolated categories, obscuring how risks emerge across workflows, systems, and time. As a result, incident data can underestimate the prevalence and significance of digital hazards, reinforcing the illusion that systems are safer than they actually are (42, 43).

This limitation reflects a deeper misalignment between the design of reporting systems and the nature of digital risk. While reporting frameworks are oriented toward discrete, attributable events, digital hazards often emerge as distributed processes across time, systems, and actors (34, 44, 45). As a result, the very structure of reporting systems contributes to the underrepresentation of such risks.

### Organisational assumptions of control

A further contributor to the invisibility of digital safety risk lies in organisational assumptions about system reliability and control. Digital maturity is often assessed through implementation metrics, system uptime, or compliance with technical standards, which can create a perception that risks are well managed once systems are operational (4, 46, 47).

These assumptions are reinforced by how digital maturity is measured, often through indicators such as system deployment, uptime, and compliance with technical or regulatory standards (9, 48, 49). While such metrics provide important information about system performance, they offer limited insight into how systems behave in everyday clinical use. As a result, the absence of reported incidents is frequently interpreted as evidence of safety, rather than as a potential indicator of unrecognised or unobserved risk.

This creates a form of epistemic closure, in which organisational confidence in system performance reduces the likelihood that emerging vulnerabilities are questioned, investigated, or made visible. In this way, assumptions of control can actively contribute to the persistence of hidden risk within digitally mediated care environments.

## The consequences of invisible risk

The invisibility of digital risk has far-reaching consequences. When hazards are not recognised, they cannot be addressed. Organisations may invest heavily in training, compliance, or additional layers of automation while neglecting deeper design and governance issues. Responsibility is often attributed to individuals when harm occurs because system-level vulnerabilities were never made explicit (6, 10, 50).

Invisible risks also undermine learning. Without clear signals, organisations struggle to identify patterns, prioritise interventions, or evaluate the impact of change. This contributes to cycles of surprise, reactive fixes, and recurring incidents, particularly following system upgrades, configuration changes, or workflow redesigns (6, 33).

At a broader level, invisibility fosters overconfidence. Digital maturity is equated with system stability, and the absence of reported incidents is interpreted as evidence of safety. This complacency delays investment in monitoring, resilience, and organisational learning precisely where they are most needed (31, 36).

## What needs to change: making digital risk visible

If digital safety problems remain invisible, improving safety requires a shift in perspective. Rather than focusing exclusively on preventing discrete failures, organisations must learn to recognise weak signals of system stress. This includes attending to near misses, workarounds, and informal adaptations, not as nuisances to be eliminated, but as indicators of underlying vulnerability (51).

Incident reporting systems should be complemented by other forms of learning that capture process, context, and experience. Narrative accounts, multidisciplinary review, and longitudinal analysis can help surface patterns that are invisible to metrics alone. Importantly, making risk visible is not a technical task alone; it is an organisational and cultural challenge that requires openness to uncomfortable insights (6).

## Discussion

### Invisibility and the limits of current safety approaches

The persistence of invisible digital safety risk challenges some of the most common assumptions about how safety is achieved and demonstrated in digitally mediated healthcare. When risks do not manifest as discrete, reportable events, they are less likely to be recognised, prioritised, or addressed (6). This creates a paradox in which systems may appear stable and mature precisely because their vulnerabilities remain hidden within routine work. In such contexts, safety becomes associated with technical deployment and compliance rather than with ongoing scrutiny of how digital systems behave in practice (6, 51).

### Implications for learning, accountability, and safety culture

A critical consequence of invisible digital risk is how responsibility for harm is understood and attributed. When system-level vulnerabilities remain hidden, adverse outcomes are more likely to be interpreted through the lens of individual error rather than as manifestations of broader sociotechnical conditions (39, 40). This reflects a well-established pattern in safety science, where visible actions at the point of care are privileged over less visible systemic contributors.

In digitally mediated environments, this dynamic is further amplified. Because many contributing factors, such as data inconsistencies, interface limitations, or workflow fragmentation, are not readily observable, they are less likely to be recognised during incident analysis (30, 52). As a result, responsibility may be disproportionately assigned to clinicians whose actions are visible, even when those actions were shaped or constrained by system design.

This misattribution has important implications for organisational learning and safety culture. When harm is framed primarily in terms of individual performance, opportunities to identify and address underlying system vulnerabilities may be missed (39, 51). Over time, this can reinforce a culture of blame and limit the development of more systemic approaches to safety improvement.

Importantly, the invisibility of HIT-related risk is not only a problem for clinical work or organisational management, but also for research and policy. Safety problems that are difficult to observe, classify, or measure are less likely to be studied, published, or incorporated into standards and guidance (6). Research approaches that privilege readily observable outcomes may therefore reinforce blind spots in the evidence base, shaping improvement efforts around what is visible rather than what is consequential. Addressing digital safety requires greater reflexivity about how knowledge of risk is produced, as well as openness to signals that fall outside conventional definitions of incidents or harm (6, 51).

These observations suggest that invisibility is not merely an empirical challenge but a fundamental epistemic limitation in how digital safety is understood and governed. When risks are not readily observable, they are less likely to be measured, reported, or prioritised, shaping both organisational learning and the broader evidence base (9, 10, 44, 53). This reinforces a cycle in which what is visible becomes actionable, while what remains hidden continues to accumulate without being addressed.

Recognising invisibility as a defining feature of digital risk shifts the focus of safety improvement. Rather than asking only how to prevent failure, it invites attention to how risks are concealed, normalised, and revealed over time (6, 51). Making digital safety risks visible is not a matter of adding more controls or metrics alone, but of developing sociotechnical awareness across design, implementation, governance, and everyday use. Without such awareness, digital healthcare will continue to fail quietly, with lessons learned only after harm becomes impossible to ignore (54, 55).

### A sociotechnical perspective on digital safety

A sociotechnical perspective is essential for understanding why digital safety risks remain invisible. Health information technologies do not operate in isolation but are embedded within complex systems of people, workflows, organisational structures, and material environments (51, 52). From this perspective, safety cannot be understood solely in terms of technical performance, but must be examined in relation to how technologies shape and are shaped by clinical practice.

A purely technical view of digital safety might focus on system functionality, uptime, or the absence of software faults. However, a sociotechnical lens reveals how risks can emerge even when systems are functioning as designed (29, 55). For example, a laboratory result may be correctly generated and transmitted within a system, yet remain effectively invisible in practice because it is displayed in a separate interface, not integrated into clinical workflows, or not aligned with clinicians' expectations of where and when information should appear. In such cases, no technical failure has occurred, yet the risk arises from the interaction between system design, workflow integration, and human cognition (29, 55).

This example illustrates how invisibility is produced not by isolated technical faults, but by the alignment, or misalignment, between technological systems and the contexts in which they are used. Recognising digital safety as a sociotechnical phenomenon shifts the focus from identifying discrete system failures to understanding how risks are distributed, normalised, and rendered invisible across everyday practice. Without such a perspective, efforts to improve digital safety may continue to overlook the conditions under which harm emerges, addressing symptoms rather than underlying system dynamics.

The relevance of these mechanisms may be further amplified in the context of artificial intelligence (AI)–enabled healthcare. AI systems often operate through complex, opaque models that can obscure how outputs are generated, increasing abstraction and reducing the visibility of underlying processes (56, 57). In such settings, information fragmentation, overreliance on automated outputs, and diffuse accountability may become more pronounced, making it even more difficult to recognise how risks emerge and propagate. As AI becomes more embedded in clinical decision-making, understanding and addressing the invisibility of digital risk will be increasingly important.
